# Producing Heavyweight High-Performance Concrete by Using Black Sand as Newly Shielding Construction Material

**DOI:** 10.3390/ma14185353

**Published:** 2021-09-16

**Authors:** Khaled A. Eltawil, Mohamed G. Mahdy, Osama Youssf, Ahmed M. Tahwia

**Affiliations:** 1 Department of Structural Engineering, Faculty of Engineering, Mansoura University, Mansoura 35511, Egypt; mmahdy@mans.edu.eg (M.G.M.); osama.youssf@mymail.unisa.edu.au (O.Y.); atahwia@mans.edu.eg (A.M.T.); 2 Science, Technology, Engineering and Mathematics Unit, University of South Australia, Adelaide, SA 5095, Australia

**Keywords:** heavyweight high-performance concrete, black sand, fly ash, fire resistance, magnesium sulfate, shielding radiation

## Abstract

Experimental work was carried out to study new fine aggregate shielding construction materials, namely black sand (BS). The BS effect on the mechanical, durability, and shielding characteristics of heavyweight high-performance concrete (HWHPC) was evaluated. This study aimed at improving various HWHPC properties, concertedly. Fifteen mixtures of HWHPC were made, with various variables, including replacing 10% and 15% of the cement with fly ash (FA) and replacing normal sand by BS at various contents (15%, 30%, 45%, 60%, 75%, and 100%). The test specimens were subjected to various exposure conditions, including elevated temperatures, which ranged from 250 °C to 750 °C, for a duration of 3 h; magnesium sulfate (MS) exposure; and gamma-ray exposure. The effects of elevated temperature and sulfate resistance on concrete mass loss were examined. The results revealed that BS is a promising shielding construction material. The BS content is the most important factor influencing concrete compressive strength. Mixes containing 15% BS demonstrated significantly better strength compared to the control mixes. Exposure to 250 °C led to a notable increase in compressive strength. BS showed a significant effect on HWHPC fire resistance properties, especially at 750 °C and a significant linear attenuation coefficient. Using 10% FA with 15% BS was the most effective mixing proportion for improving all HWHPC properties concertedly, especially at greater ages.

## 1. Introduction

Highlights

- Black sand is a promising shielding construction material.
- HWHPC with BS is suitable for NPP construction.
- BS improved the sulfate and fire resistance of HWHPC, especially at 750 °C.
- Using a high content of BS had an inverse effect on various HWHPC properties.
- HWHPC with 15% BS and 10% FA had significantly improved properties.

Black sand (BS) is a high economic and strategic value sand, as it contains many economically useful radioactive minerals such as uranium, zircon, hafnium, and thorium, and heavy non-radioactive materials (iron, ilmenite, rutile, granite, heavy silica, monazite, titanium, and other minerals). Although it is used in various industrial applications, such as the nuclear industry or other metallurgical and engineering industries [1], it has not yet been used as a shielding construction material.

It is a dense mineral, with a specific density greater than 2.85. It can be classified into two main types: dark BS and diluted BS. Dark BS can be found in the western parts, toward the west of Rosetta, Egypt, with various heavy minerals, which when its percentage in sand increases up to 90%, gives it a black color. The diluted BS, which is lighter in color, ‘varies from greyish to yellow’ and can be found in central areas among the Rosetta and Damietta outpourings, and in eastern part toward the east of Damietta [2,3]. It contains up to 40% heavy constituents. 

Various radiation-shielding materials are beneficial to avoid the harmful influences of radiation on people and the environment. These materials vary in their efficiency, according to the type of radiation to be absorbed. Heavy materials such as steel, lead, lead glass, polythene, and concrete are appropriate for attenuating radiation [4,5]. Concrete is a construction material and is widely used for reactor protection due to its relatively low cost, appropriate mechanical characteristics, maintenance efficiency, and structural and production effectiveness [6,7]. It is designed to resist various ranges of load and environmental exposures. All concrete types are good radiation shielding materials; however, their structural element thickness differs with their utilized type efficiency [8].

The increasing demand for electric power has led to an increase in nuclear power plants (NPPs). The construction of NPPs is subject to many kinds of loading phenomena during their life, from the load of gravity to the load of accidents, such as earthquakes, blast loads, impact loads from tornadoes or projectiles, and thermal stress (from high temperatures). This results from atomic reactions and exposure to various radiation, as well as corrosion of the concrete structure from exposure to salts of sulfates and chlorides. To prohibit the negative impact of these radiations on people and the environment, shielding concrete must be high performance, high strength, and high hardness, with low permeability to air and water. 

Heavyweight concrete (HWC) is a certain form of concrete that is used to shield NPPs, laboratories, sterilization, radiotherapy rooms for transmission, store radioactive wastes, and hospitals for ionizing radiation shields, due to its low cost, ease of maintenance, environmentally friendliness, durability, satisfactory mechanical properties, and shielding attenuation properties [6,7]. HWC has the same components as normal concrete (NC), but HWC uses particular kinds of aggregates with high gravity as fillers for the alleviation of γ-rays or neutrons sequentially. It is typically a combination of hydrogen and other light nuclei, and also nuclei with a temperately high atomic number [9]. Owing to its intrinsic water and high intensity, it is used as a superior material for shielding radiation, combined with its shielding characteristics for the rays of neutrons and gamma [10,11]. Therefore, heavy components and high atomic weight components are essential in RSC [12].

To produce HWC with such features, several factors are taken into consideration, such as cost, availability of shielding materials, and radiation type, before selecting appropriate shielding materials. HWA is an essential constituent material in HWC mix design. It contains a large component of material from the metallic stages with high atomic bloc, such as ilmenite, hematite, barite, or magnetite, which function well in enhancing HWC properties, particularly shielding properties [13,14]. These metallic stages of concrete are significant in making it efficient in attenuating the fast neutrons [15,16,17,18,19]. In other words, HWC’s properties rely upon the properties, types, and quantity of the utilized HWA [20]. The physical and chemical characteristics of the HWA have a great influence on the adhesion and absorption at the aggregate–paste interface and the shielding properties for photons and neutrons [13,14]. HWC has a considerably higher bulk density than NC. HWC has a greater bulk density (≥2900 kg/m^3^) than NC [21]. 

The radiation shielding characteristics are the essential advantages of HWC materials [22,23]. Nevertheless, the elevated strength of the HWC creates the additional quality of decreasing the thickness of the concrete construction, while preserving the required strength load-bearing over the NC. A previous study noted that using HWC as a nonstructural substance facilitates the decreasing of wall thickness by approximately 40% compared with walls produced using ordinary concrete [24].

Numerous investigations have been conducted to study the impact of various aggregate types on HWC properties, particularly the attenuation properties. It was argued that aggregates containing a high atomic number such as ilmenite, hematite, barite, limonite, lime, magnetite, galena, lead, and steel slag are suitable as anti-radiation materials against gamma rays [25]. Other studies showed that concrete penetration by neutrons can be reduced by using elements that contain a low atomic number and high bound water and boron in their structure [26,27]. 

Many methods are used for producing HWC. First, replacing natural sand, gravel, or both with HWA whose bulk density exceeds 4000 kg/m^3^, according to BS EN 206-1 [28]. Second, using a special cement type, supplementary cementing materials (SCMs), and chemical admixture to enhance the concrete microstructure. Third, using both previously mentioned techniques. The utilization of waste products, especially SCMs, is a successful method for developing HWC. Moreover, it decreases energy usage [29,30,31]. If SCMs are added to cement, it will dramatically increase the advancement of the concrete industry, because of the savings in costs and energy, the protection of the environment, and the preservation of resources [32]. 

Fly ash (FA) is a by-product of coal-fired processes and can be used as an SCM for the production of HWC. It is rich in silica and alumina elements [33], and when using it in concrete it has a morphologic effect, pozzolanic effect, and micro-aggregate effect. Despite its slow rate of reaction, FA significantly improves concrete workability and long-term strength. FA has a function in the production of concrete with low permeability [34]. The ACI should take caution when utilizing SCMs, to accomplish a suitable heavy density of concrete [35].

Using fine materials is another successful method for filling the voids in concrete to improve the properties of HWC. This decreases pore size and porosity. The relationship between radiation and absorbent materials is enhanced by fine materials with a high specific surface area [36]. The filler enhances the compact packing of the microstructure. As a consequence, the porosity is minimized, which improves the radiation shielding performance [37]. Ouda [38] developed high-performance heavy-density concrete utilizing a fine magnetite aggregate and stated that it improved the shielding performance against gamma radiation.

## 2. Research Objectives

To the best of the author’s knowledge, no research has been carried out to date on utilizing BS as a novel construction shielding material in concrete. Furthermore, there are no standards or criteria for analyzing the effect of BS on concrete performance. This research presents the first fundamental investigation of the performance of HWHPC made of BS and FA being exposed to elevated temperature, sulfate, and radiation. This research will advance the structural applications of power stations, particularly NPPs.

## 3. Methodology of Research

This study investigates the influence of BS on the characteristics of heavyweight high-performance concrete (HWHPC). There were fifteen HWHPC mixes produced, varying in BS, PC, FA, and W/B ratio. NS was substituted by various BS replacement ratios, varying from 0% to 100% to produce HWHPC. Two partially FA replacement proportions of 10% and 15% were used. To ensure that the used W/B was not affected by concrete properties and appropriate for a specific surface that differs with varying BS ratios, several trials were performed in each concrete group, using a flow table slump test. Furthermore, aggregates in the condition of a saturated surface dry (SSD) were used to prevent the effect of aggregate water absorption during mixture, to assess the true effect of the aggregate on the concrete properties. 

### 3.1. Materials

**Figure 1:**
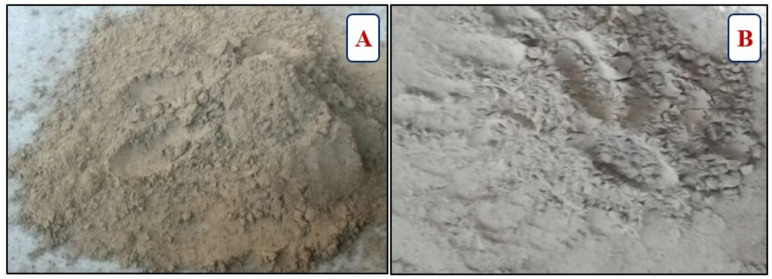
Used binder material: (**A**) Portland Cement, (**B**) Fly Ash.

**Figure 2:**
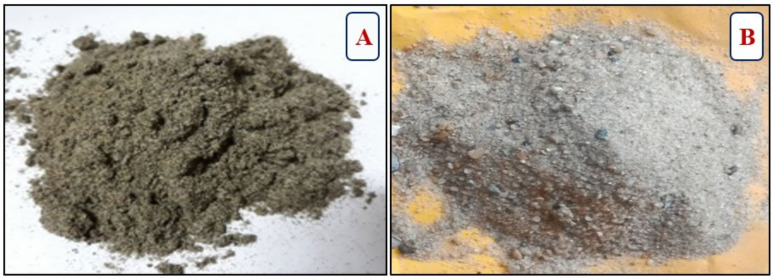
Used fine aggregate: (**A**) Black Sand and (**B**) Normal Sand [41].

**Figure 3:**
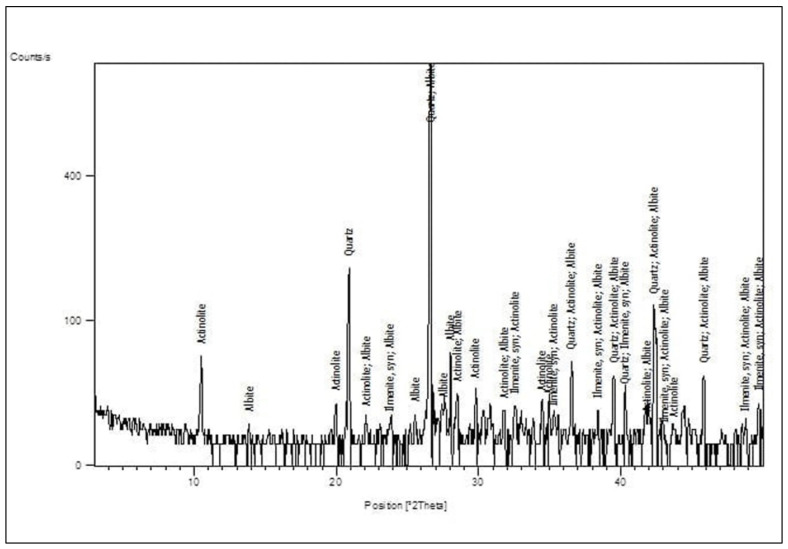
Black sand XRD analysis [41].

**Figure 4:**
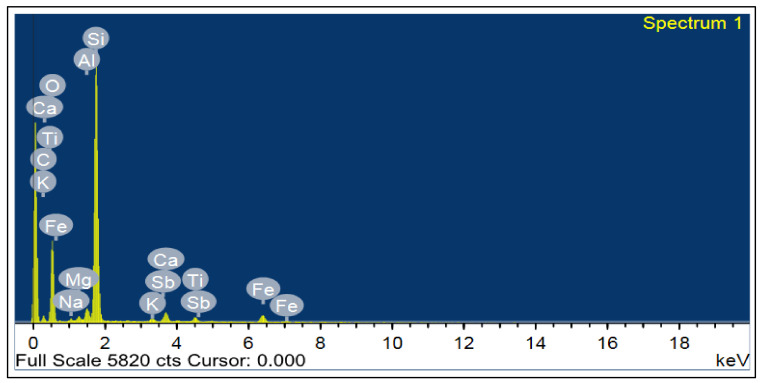
Black sand EDX analysis [41].

**Figure 5:**
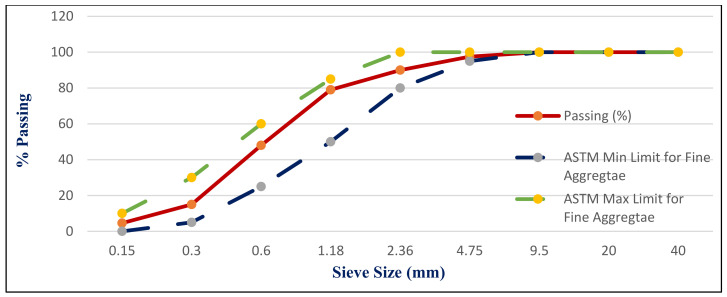
Normal sand grading [41].

**Figure 6:**
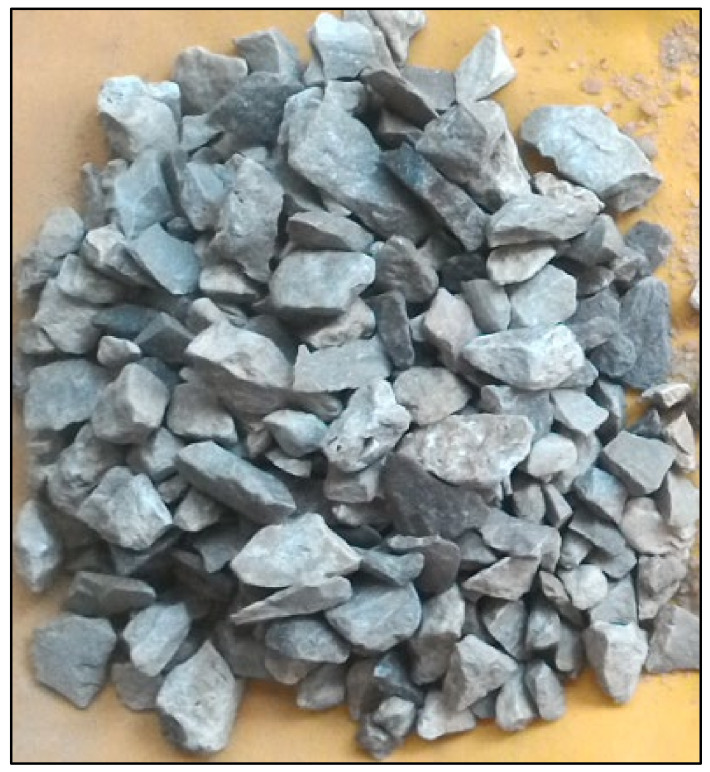
Used Crushed Dolomite.

**Figure 7:**
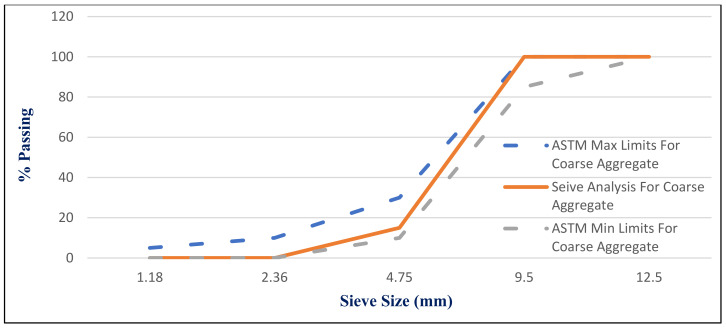
Dolomite grading curve.

CEM I 42.5 N Portland cement (PC) type according to BS EN 197-1:2011 [39] with a specific gravity of 3.2 was adopted as the binder material in this study, as shown in Figure 1A. Fly ash, class F, with a specific gravity of 2.2 was used at 10% and 15% as cement replacement, as shown in Figure 1B. Superplasticizer type G, according to ASTM C-494 [40], with a specific gravity of 1.08 was added to all mixtures. Greyish-yellow type BS from the Baltium shore, Egypt, with a specific gravity of 2.65 was used in all mix sets, except the control set. Figure 2A shows the BS used in this study [41], and the BS had particle sizes between 0.15 and 0.3 mm, with a 2.2 fineness modulus and 92 cm^2^/gm surface area. Figure 2 and Figure 3 show the analysis of X-ray Diffraction (XRD) and energy-dispersive X-ray spectroscopy (EDX) for BS used, as illustrated in Figure 3 and Figure 4 [41]. The NS used had a fineness modulus of 2.9 and a specific gravity of 2.55, as shown in Figure 2B. Its grading sieve analysis is shown in Figure 5 [41]. Dolomite with a maximum nominal size of 12.5 mm was utilized as coarse aggregate, as shown in Figure 6. Figure 7 presents the used dolomite sieve analysis results. The aggregate was cleaned in the sieve to eliminate any harmful materials and chloride contamination. Table 1 displays the chemical composition of the binder materials and BS [41].

### 3.2. Mix Proportion

In this study, NS was substituted by various BS replacement ratios, varying from 0% to 100%, to produce HWHPC. Fifteen HWHPC mixes were carefully designed, according to absolute volumes. The absolute volume approach is widely accepted and is considered to be more convenient for HWHPC. It was thus used to obtain a denser concrete. To guarantee that the used W/B did not influence HWHPC properties and therefore was suitable for a specific surface with varying BS ratios, the W/B ratio for each mixture was evaluated through flow table slump test trial and error. All HWHPC mixes, thus, had a constant SP to a cement ratio of 1.5%, and the water was used to maintain a flow table slump diameter of 20 ± 2 cm. To examine the influence of the SCMs on the characteristics of concrete comprising BS, two sequences of HWHPC mixes were designed using 10% FA and 15% FA as a proportional addition to PC.

The used binder had a content of 500 kg/m^3^. The mixes were categorized into 3 groups: control, Z, and V. There was no BS in the control group mixtures. X0, Z0, and V0 are control mixtures without FA and with 10% FA and 15% FA, respectively. Groups Z and V comprised 10% and 15% FA, respectively, with BS ratios varying from zero to 100%. Table 2 lists mixed constituent proportions per 1 m^3^ of the concrete mix.

### 3.3. Mixing, Curing, and Testing 

The mixing process for HWHPC was identical to that for NC. In a standard mixing process, the materials were blended in the correct sequence: coarse and fine aggregate were briefly mixed dry for 2 min for each mix, accompanied by PC blended with FA. Upon adding 80% of the mixing water, the mixer was turned on. After 1.5 min of mixing, the remaining water was gradually added to the mixer. For a total of 5 min, all batches were mixed.

**Figure 8:**
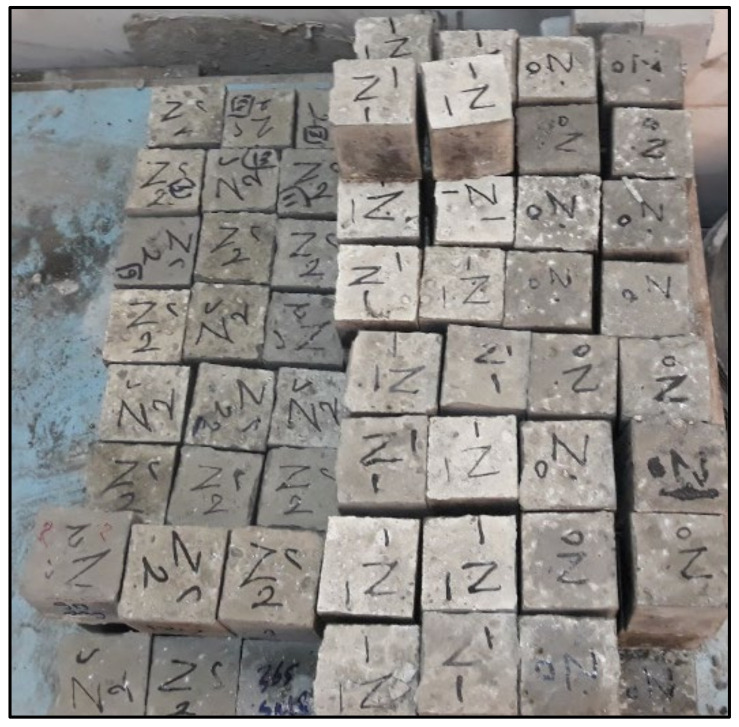
Group Z samples.

All concrete samples were cast in three layers into 70 mm × 70 mm× 70 mm waterproof wood cubes with compacting of each layer using a vibrating table. The concrete specimens were sealed with a plastic membrane after casting to prevent water evaporation. After de-molding, the specimens were submerged in a water tank until the testing day. As a result, sample curing was carried out in compliance with ASTM C511 [42]. Figure 8 shows the group Z samples. The durability of NPP concrete is primarily determined by the resistance to external harmful agents such as sulfate attack, elevated temperature, and radiation. The research relied upon the following tests as the primary measure to assess the viability of using BS in HWHPC.

#### 3.3.1. Density of Concrete

ECP 203-2020 was used to determine the density of the hardened concrete [43].

#### 3.3.2. Compressive Strength

**Figure 9:**
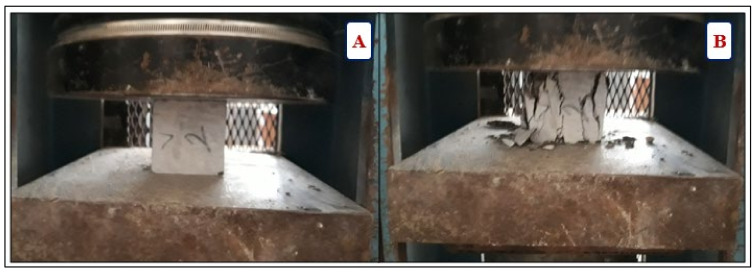
Compressive strength test: (**A**) test setup, (**B**) specimen failure.

The compressive strength of concrete was evaluated at 7, 28, 90, and 180 days. The test was carried out on three 70-mm cubes per measure using a 2000 kN testing machine with a loading rate of 0.6 MPa/s. Figure 9 illustrates the compressive strength of the V2 sample before (A) and after (B) testing.

#### 3.3.3. Effect of Elevated Temperature Exposure 

**Figure 10:**
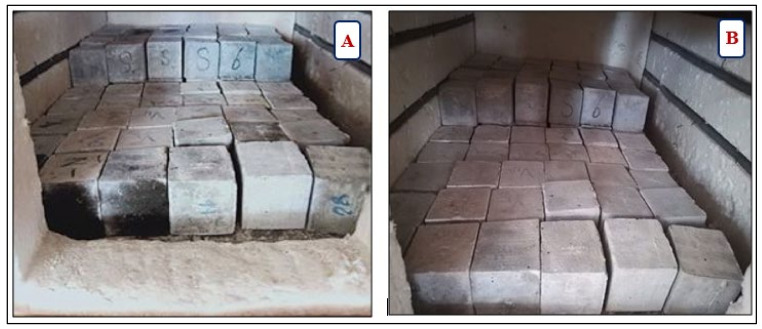
Concrete samples before (**A**) and after (**B**) 500 °C fire exposure.

**Figure 11:**
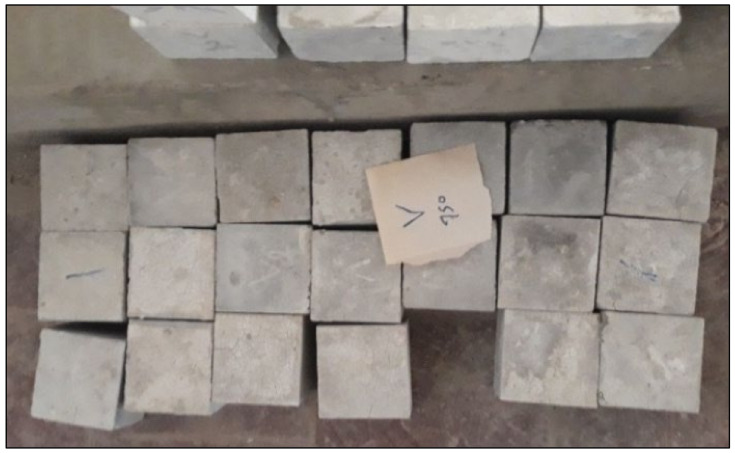
Group V samples after exposure at 750 °C.

Mass loss, scanning electronic microscopy (SEM), energy-dispersive X-ray spectroscopy (EDX), and residual compressive strength determination were used to characterize the HWHPC behavior under thermal gradients. To prevent spalling, 15 standard curing aged cubic specimens of 70 mm were dried at 100 °C in a furnace till reaching constant weight and then exposed to one of the temperature gradients (250 °C, 500 °C, and 750 °C) for 3 h. The heating process was performed in a laboratory furnace at room temperature (20 °C) and progressively raised at a rate of 6.7 °C/min until the setpoint temperature was reached. Figure 10 shows the sample before (A) and after exposure at under 500 °C for 3 h (B). Figure 10 and Figure 11 displays the group V samples after exposure at 750 °C. 

After the firing time was completed, the specimens were cooled down for 1 h by opening the furnace door, followed by another 23 h at laboratory room temperature until testing. To distinguish any changes during the test phase, the original mass and compressive strength of the specimens were recorded prior to implementing the elevated temperature. Equation (1) was used to observe the alteration in the block for each sample:

(1) $$\mathrm{Mass}\;\mathrm{Loss}\;\left(\Delta \mathrm{Mf}\right)=\frac{M_{x0}-M_{ft}}{M_{x0}}$$

where Mass Loss is change of mass at time *t* (%), *Mft* is the specimen mass (g) under fire exposure temperature (t) at 180 days. *Mx0* is the specimen mass (g) with normal water curing at 180 days.

#### 3.3.4. Magnesium Sulfate Exposure 

A series of tests were proposed to determine the HWHPC resistance to magnesium sulfate (MS). The effect of MS on HWHPC samples was continuously monitored at frequent intervals throughout the exposure period, using various tests, such as weight change, residual compressive, and SEM. The procedures involved the ability to immerse three specimens per mix in a water solution comprising 5% MS (equal to 33,800 ppm SO_4_^−2^) and updating the solution monthly.

Compressive strength was measured at 28 days as an initial strength and then at 90, 180, and 365 days in a sulfate solution. For weight change, X0 was first primed in MS for various timepoints and their weight in saturated surface dry conditions was recorded as the initial weight. Prior to the weight measurement, samples were tested from the sulfate solution and were wiped clean. Specimens were measured during the exposure period and the rate of weight change was calculated as a percentage of the initial weight. The mass change for each sample was determined using Equation (2).

(2) $$\mathrm{Mass}\;\mathrm{Loss}\;=\frac{Mx0-Mst}{Mx0}$$

Where Mass Loss is change of mass at time *t* (%), *Mst* is the specimen mass (g) under MS exposure at time t days. *Mx*0 is the specimen mass (g) under normal water curing at 90 days.

#### 3.3.5. Radiation Attenuation Test

**Figure 12:**
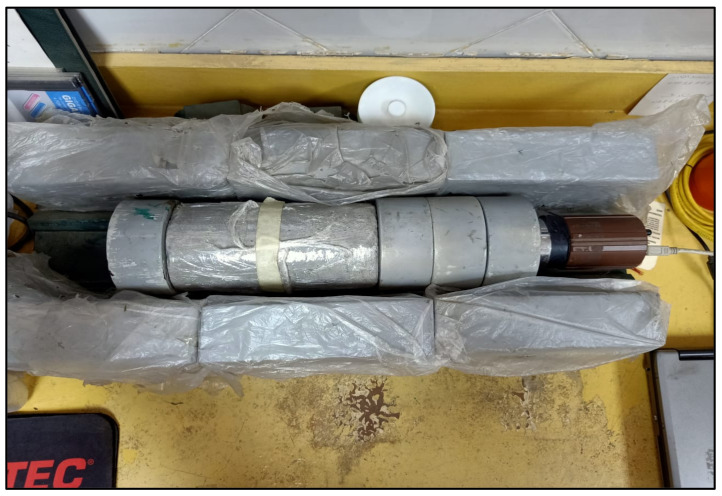
Attenuation shielding test.

Radiation shielding tests were carried out on the specimens of HWHPC, to assess their radiation shielding efficiency using gamma-rays (γ-rays) from Caesium-137 (^137^Cs), which emits 0.66 MeV photons. The isotope decay rate for ^137^Cs used in this analysis was 10.0 mCi. Specimens were extracted and were dried in an oven at 105 °C before the examination, as recommended by [44]. The counter was 300 mm away from the gamma radiation source. The cylindrical concrete specimens were tested immediately after curing for 28 days. Concrete cylinder height and diameter were 150 and 300 mm, respectively. The exposure of the examined specimens to gamma rays was for 20 min. Figure 12 shows the performance of the shielding attenuation test for sample Z1. 

A material’s radiation shielding properties are revealed in the coefficient of linear attenuation (μ), which is shown as:

N_x_ = N_o_ × e^−μx^ (3)

where N_x_ is the background number of recorded counts in the detector without the HWHPC specimen, which is between the detector and the source. N_o_ is the background number of counts that are recorded in the detector with the HWHPC specimen between the detector and the source, and X refers to the thickness of the HWHPC specimen. 

The half-value layer (HVL) and tenth-value layer (TVL) are absorber thicknesses that decrease the intensity of gamma radiation to a half and tenth, respectively. These can be estimated using the following equations:

X_1/2_ = ln × 2/μ (4)

X_1/10_ = ln × 10/μ (5)

The mean free path (mfp) is defined as the average distance between two successive photon interactions and is expressed as:

mfp = 1/μ(6)

## 4. Results

### 4.1. Density

**Figure 13:**
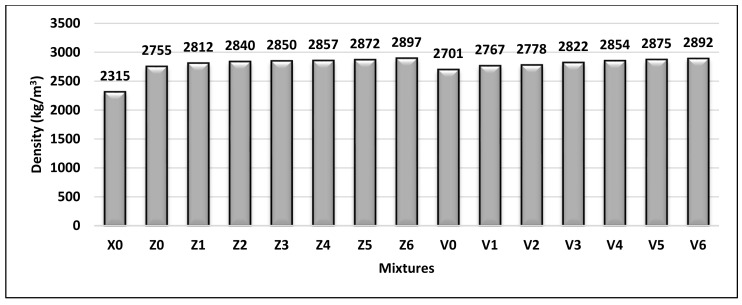
Hardened density of all mixtures (kg/m^3^).

The density of hardened concrete in this study was measured and is plotted graphically in Figure 13. The results showed that all mixes achieved a density of more than 2600 kg/m^3^, so they are classified as HWC according to BS EN 206-1 (2013) [28]. In general, the concrete density was relative to the increased BS content. Whereas, the mixes that have BS as fine aggregate along with 10% FA (Z mixes) and 15% FA (V mixes) were found to be slightly higher in density than X0, by 19–30% and 17–25%, respectively. The group Z mixes had a density slightly higher than that of group V, due to the higher FA content in the group V mixes.

### 4.2. Mass Loss

#### 4.2.1. Effect of Elevated Temperature Exposure

**Figure 14:**
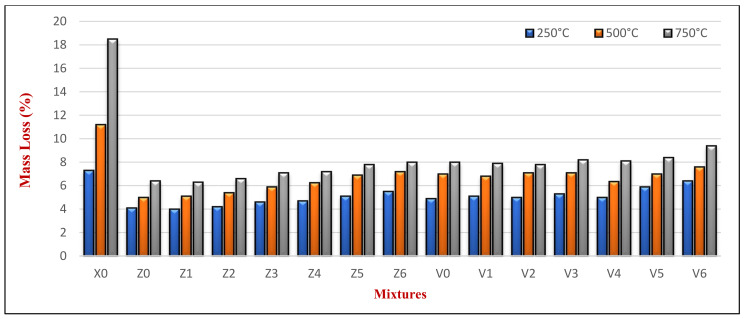
HWHPC mass loss (%) under fire exposure.

Figure 14 presents the average mass loss (ΔMf)% due to various elevated temperature exposures at 180 days, resulting from the water evaporation from HWHPC. The results in Figure 14 are the average of the three cubes tested per mix. The loss of mass increased with increasing the temperature for all the samples at 180 days. The Z and V mixes experienced an average ΔMf of 4.6% and 5.37% at an elevated temperature of 250 °C, 5.69% and 6.99% at an elevated temperature of 500 °C, and 7.05% and 8.25% at an elevated temperature of 750 °C, respectively. These are compared to the 7.3%, 11.2%, and 18.5% for X0 at an elevated temperature of 250 °C, 500 °C, and 750 °C, respectively.

The mass loss was due to the evaporation of water more than the decomposition of hydrate in the concrete. It is well known that there are many compositions of water in concrete; including, capillary water, water that is absorbed physically, and water that is bound chemically in CSH and CH [45]. The first and second types of water are most of the water formed in concrete. 

At 250 °C, the first water evaporates and the second water is lost gradually, leading to a great mass loss in the concrete. At 250 °C, the loss of mass in the concrete is due to the hydrates dehydration reaction, and the CH and ettringite (AFt) begin to spoil [46]. Due to the release of bound water from the cement paste, voids of air are formed in the concrete, causing a mass loss at 500 °C. This results from the whole or partial deterioration of CH, Aft, and CSH. The mass loss of concrete at 750 °C is because of the continuous decomposition of CSH [47]. Producing a dense structure from HWHPC prevents the escape/evaporation of water from the concrete pores.

This study found the loss of mass increased when the temperature increases accordingly, which indicates the decomposition of the constructional integrity of the concrete. The gradual advancement of the voids in the specimens was also evidence in the case of evaporation. Figure 14 shows that the loss of mass of group Z and V mixtures was smaller than that of X0 (containing natural aggregate). Moreover, all mixtures containing BS up to 60% showed a constant mass loss compared to their control group mixture (for example, mixtures Z1, Z2, Z3, and Z4 compared to Z0). However, Z5 and Z6 showed a higher mass loss than Z0. This may have been related to the FA and BS mineral crystalline changing at high temperatures over 500 °C, such as iron and quartz [48].

#### 4.2.2. Magnesium Sulfate Exposure 

**Figure 15:**
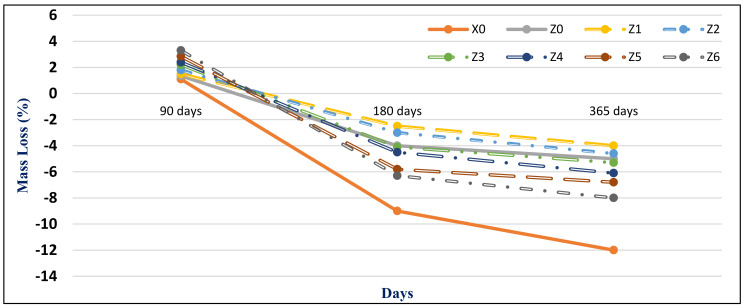
Group Z mass loss (ΔMs %) due to magnesium sulfate exposure at various curing ages.

**Figure 16:**
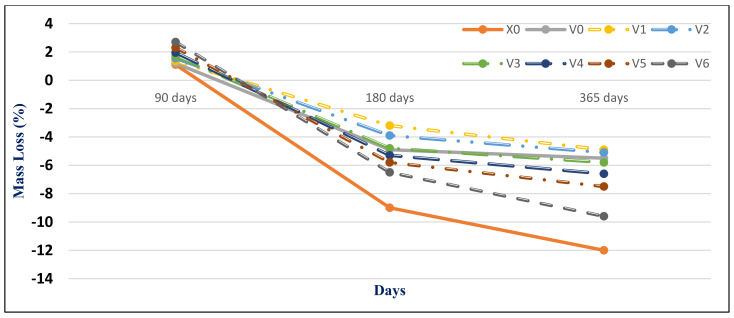
Group V mass loss (ΔMs %) due to magnesium sulfate exposure at various curing ages.

**Figure 17:**
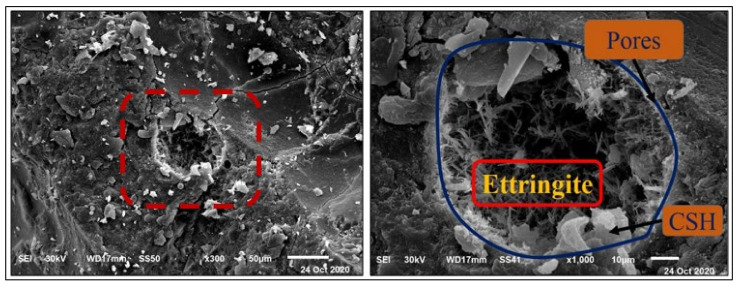
Ettringite formation and pore filling in Z0 concrete at 90 days.

Figure 15 and Figure 16 present the HWHPC mass loss ratios for groups Z and V at different exposure ages, respectively. All HWHPC showed a slight mass gain at 90 days compared with the control mixture X0, due to the development of unstable HWHPC microstructure crystal ettringite, which filled the pores at 90 days, as shown in Figure 17. The mass loss increased at 180 and 365 days, due to deterioration of the microstructure as the ettringite expanded at 180 days [49].

### 4.3. Compressive Strength

**Figure 18:**
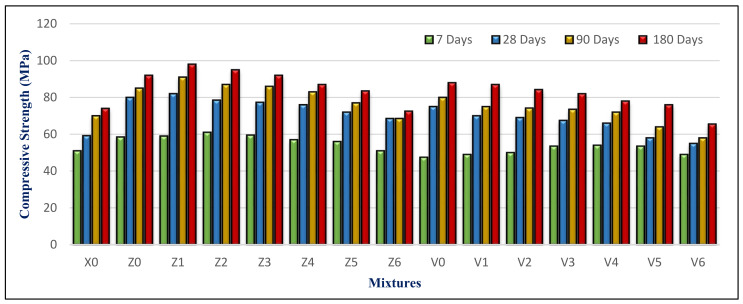
Compressive strength results at various time points.

**Figure 19:**
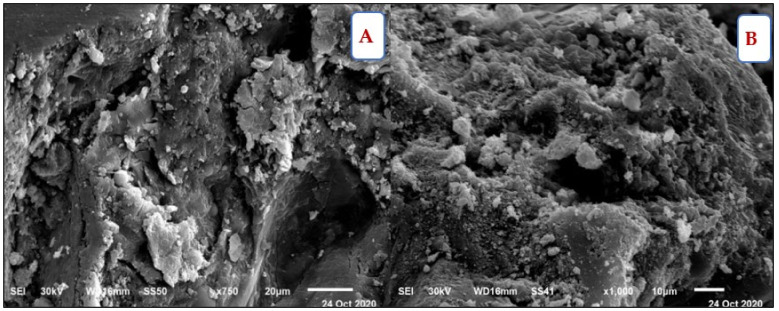
(**A**) Z1 microstructure SEM at 180 days under normal conditions, (**B**) Z6 microstructure SEM at 180 days under normal conditions.

Figure 18 depicts the compressive strength for all mixes at 7, 28, 90, and 180 days. The BS content is the most important factor influencing this. It also was inversely related to the BS content. Mixes containing BS with a substitution ratio ranging from 15% to 60% demonstrated a constant strength as compared to control mixes containing FA but no BS. It did, nevertheless, exceed the X0 in compressive strength, particularly at later ages. This is attributed to the BS filler effect. Using a suitable proportion of BS blocked pores and capillary pores in the microstructure. Therefore, it becomes denser and compacted, as in the Z1 microstructure shown in Figure 19A. 

Mixes Z1 and V1 had the greatest strength of the groups. Mix Z1 with 15% BS and 10% FA had compressive strengths of 58, 82, 91, and 98 MPa at 7, 28, 90, and 180 days, which was the highest among the mixes. The average strength advancement in HWHPC systems is based on the pozzolanic activity of FA as the mineral mixture, and the physical and mechanical characteristics of the utilized BS content as fine aggregate, in which the compressive strength has an inverse relationship with the BS content. 

The compressive strength of HWHPC mixes increases with curing time for all hardened mixes. This is due to increasing the hydration products (especially the tobermorite gel). Its development is due to its efficiency and the mineral content, which presents an impact on the filler and a pozzolanic response. Consequently, this leads to purring refinement by consumption of the weakened binder of calcium hydroxide (CH), with the composition of a strengthened calcium silicate hydroxide (CSH) binder.

**Figure 20:**
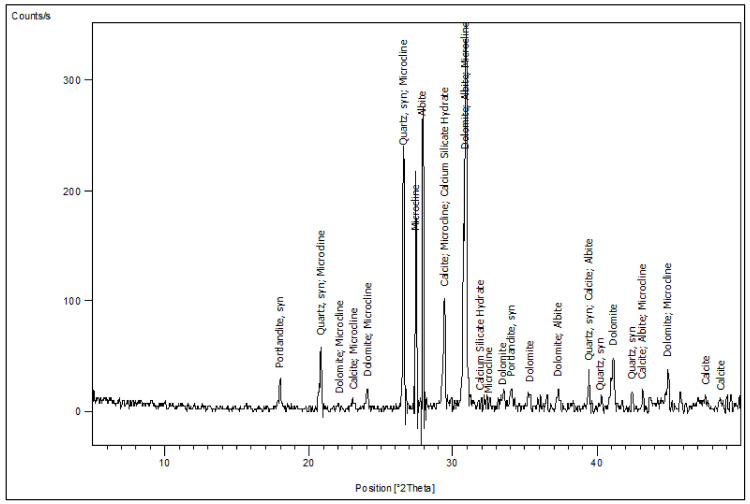
Z6 XRD analysis.

Although the FA content increased in the group V mixture, the compressive strength was lower than the group Z mixtures, which contained the same BS proportion ratio. This is attributed to an increase in the concentration of minerals such as titanium and aluminum from FA and BS in the mixture. Increasing the content of BS over 60% had an inverse effect on the compressive strength, because of generating pores that weakened the concrete microstructure, as in the Z6 microstructure shown in Figure 19B. Furthermore, the voids formed next to the porous interfacial transition zone (ITZ) may have generated a weakened bond between the coarse aggregate and mortar matrix. The Z6 XRD analysis showed the formation of microcline (K(AlSi_3_)O_8_), which evidences the high concentration of Al, as shown in Figure 20. 

### 4.4. Effect of Elevated Temperature Exposure 

Elevated temperatures are among the most significant risks to buildings and infrastructure. Previous research found that a complex fire resistance phenomenon depends on various parameters, including the aggregate kind, binder form, w/b, explosion temperature, fire time, fiber presence, and the presence of humidity in the concrete [50,51,52]. This causes many problems in concrete, such as a loss of strength, spalling and cracking, and devastation of the bond between the cement paste and the aggregates in the ITZ [53].

**Figure 21:**
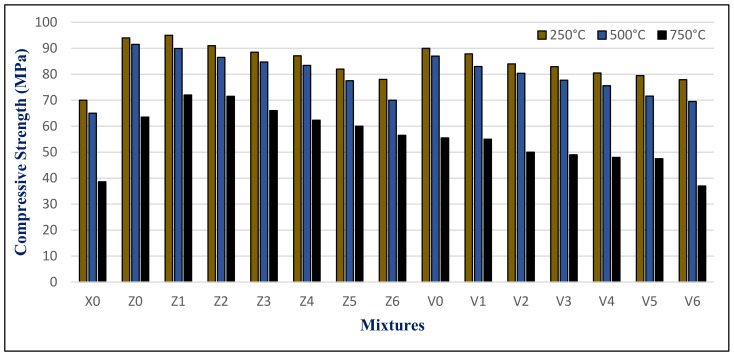
Groups Z and V strength results after exposure to elevated temperatures at 28 days.

**Figure 22:**
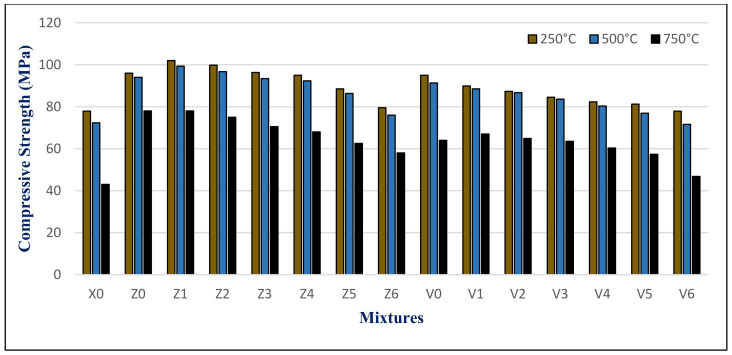
Groups Z and V strength results after exposure to elevated temperatures at 90 days.

**Figure 23:**
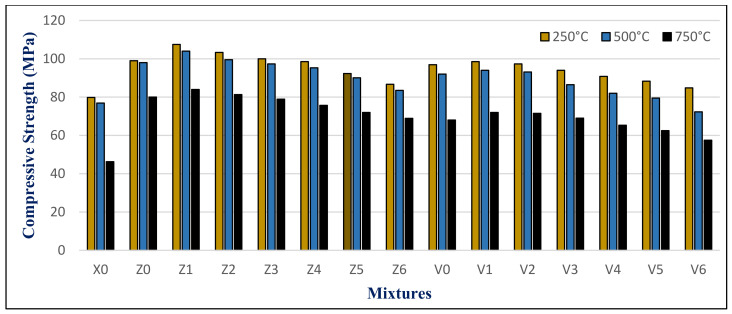
Groups Z and V strength results after exposure to an elevated temperature at 180 days.

The results of the maximum compressive strength (MPa) of the HWHPC at various degrees of temperature for a 3 h duration after 28, 90, and 180 days is graphically presented in Figure 21, Figure 22 and Figure 23. Exposure to 250 °C led to increasing the compressive strength. Then, a slight reduction in strength compared to 250 °C was recorded at 500 °C. It continued to reduce with an increase in fire temperature to 750 °C. Furthermore, it decreased with increasing BS replacement ratio through various elevated temperature exposure conditions. Even so, as the curing age increases, so does the strength. The results revealed a significant effect of BS on HWHPC fire resistance properties, especially at 750 °C.

The BS mixture strength values increased up to 15% BS for group Z at different curing periods, then decreased slightly up to a 60% BS ratio. More than 60%, the BS mixes demonstrated an extraordinary decay in strength at varying ages, particularly at 100%. Under various exposure atmospheres, the compression strength improved more than that of X0.

In group V, the BS mixture strength values decreased as the BS ratio increased. At over 60% BS, the BS mixes begin to display extraordinary drops in strength when compared to the control group mixture (V0), at various ages, and particularly at 100%. Under different exposure conditions, the compressive strength increased more than X0, except for V6.

The HWHPC mixes presented a significant residual strength gain at elevated temperatures due to appropriate minerals from BS and FA that improved the concrete microstructure. Water vapor being prevented from escaping the concrete pores was due to HWHPC ITZ being strengthened; therefore, the self-curing process began.

**Figure 24:**
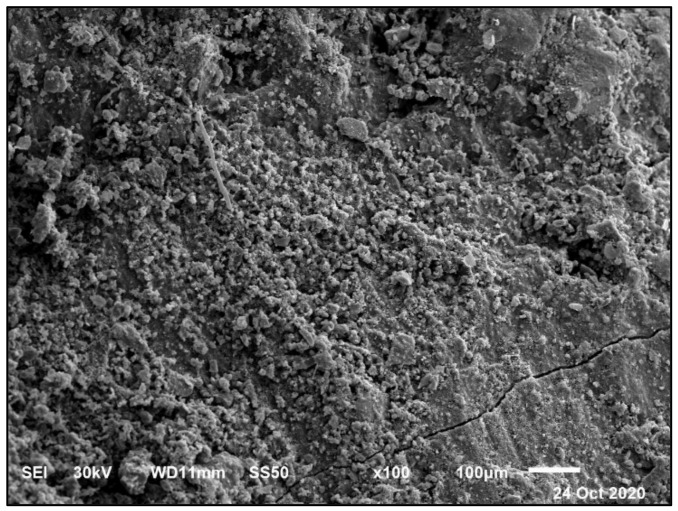
SEM of mix Z1 under 500 °C fire exposure at 180 days at a scale of 100 μm.

**Figure 25:**
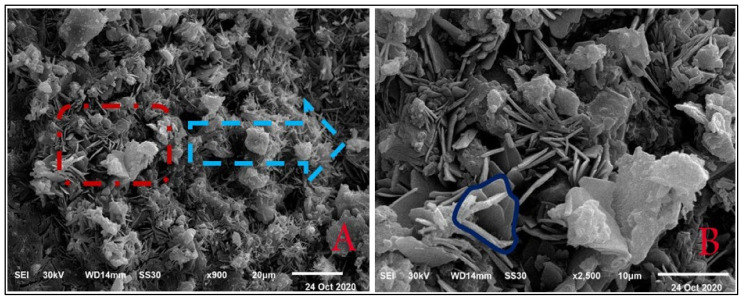
SEM of mix Z1 under 500 °C fire exposure at 180 days: (**A**) at a scale of 20 μm, (**B**) at a scale of 10 μm.

**Figure 26:**
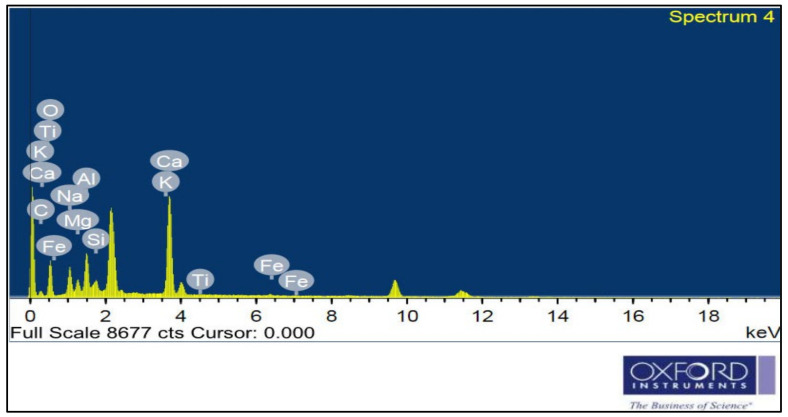
EDX analysis for mix Z1 under 500 °C fire exposure at 180 days.

That is why the strength gained at 250 °C is reflected in the mass loss results. The strength at 500 °C for the BS mixture did not decrease due to the formation of Friedel’s Salt (**FS**) in the microstructure, as in Figure 24 and Figure 25, and the analysis of EDX that is reflected in the results at 750 °C, as shown in Figure 26. It is a slight decrease compared to X0, with a significant deterioration at 750 °C. 

The FS chemical formula is 3CaO·Al_2_O_3_·CaC_l2_·10H_2_O. In cemented systems, the chloride binding ability is controlled by aluminum silicate phase (C_3_A) and C_4_AF content; chlorides such as NaCl are bound by C_3_A and C_4_AF to form FS. The latter is formed either entirely or partly by iron when aluminum is replaced or as a ferrite phase hydration element. Along with the volume expansion of ettringite (Aft), during the formation of hydrate the volume of solid-phase FS expands and is stable [54,55]. 

### 4.5. Magnesium Sulfate Exposure

In recent decades, there has been a lot of emphasis on concrete sustainability. Following reinforcement corrosion and concrete deterioration, sulfate attack is the second most important durability problem. This causes the concrete to expand, crack, lose strength, and decompose. Sulfate resistance can be influenced by several variables, such as cement type, binder component, and the sulfate cation type and concentration, as well as concrete quality, pH, W/B, and exposure period. Furthermore, MS has the fastest and most efficient influence on concrete. The effects of MS on concrete properties are more serious than those of sodium sulfate [56].

**Figure 27:**
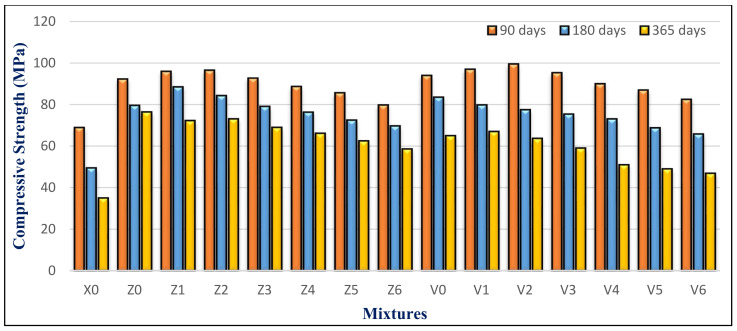
Group Z and V strength results after sulfate exposure.

The compressive strength of the HWHPC samples submerged in water and sulfate solutions at 90, 180, and 365 days is shown in Figure 27. The compressive strength decreased with increasing MS exposure time. Lower substitution levels, in ranges between15% to 45%, showed higher compressive strength for both groups. However, using 100% BS as a fine aggregate gave a poorer performance, relative to that of the other percentages. Using 10% FA, with BS in the range of 15% to 30%, was more effective for sulfate resistance than 15% FA, especially at later ages. The concrete mixtures with BS, at various storing ages in MS solutions, demonstrated a considerable improvement in compressive strength compared to the reference mixture. The compressive strength of X0 recorded a substantial degradation at 180 days, by 28.9%, compared to the same mixture at 90 days.

After 90 days of immersion in MS solutions, all mixes showed an increase in compressive strength of 5% to 16.5% and 17% to 42% for groups Z and V, respectively. Until that curing age, an increase in resistance is due to the hydration of the anhydrous cement in the sample. However, it is also due to the response of SO_4_^2^^−^ with CH to form gypsum that prevents the micro pores and supplies the structure density. It is also due to the composition of the secondary ettringite, because this stage swells greatly, leading to the densification of the hardened mixture.

The initial increase in concrete compressive strength of groups Z and V after 90 days of sulfate attack can be attributed to the following:

1. The FA pozzolanic and BS fineness effects were less porous and more dense compared to X0. In BS concrete, the entry of sulfate ions was slower than in X0.
2. The initial infusion of sulfate ions contributed by the formation of CaSO_4_ and ettringite to the filling of the micro-pores.

**Figure 28:**
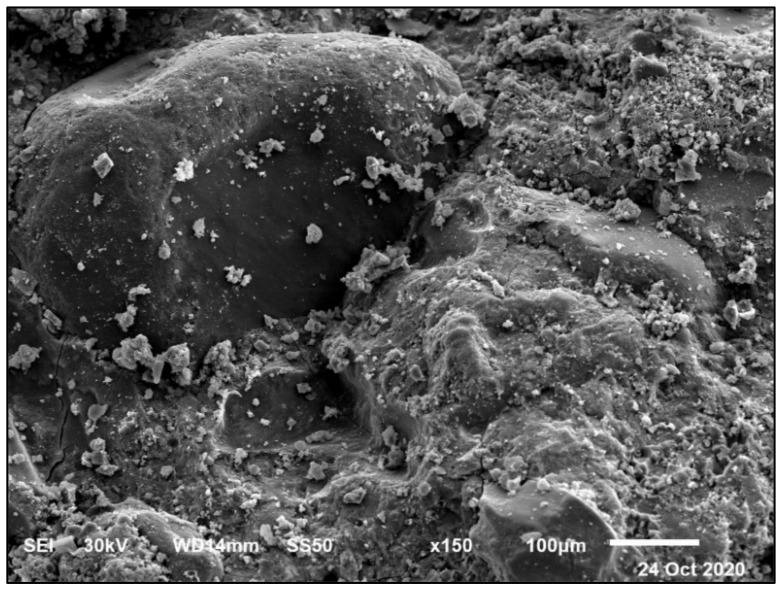
SEM of mix Z1 under sulfate exposure at 180 days at a scale of 100 μm.

**Figure 29:**
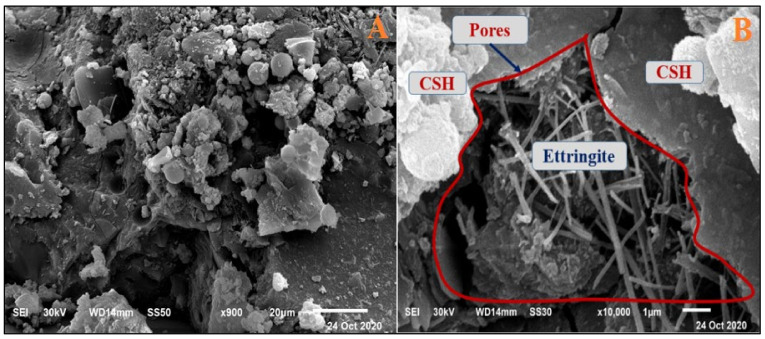
SEM of mix Z1 under sulfate exposure at 180 days: (**A**) at scale of 20 μm, (**B**) at a scale of 1 μm.

Due to previously stated reasons, the microstructure becomes thicker and denser and thereby increases the compressive strength of the BS mixtures. The decrease in the compressive strength of the control mixture in the MS solution after 90 days was because of the detachment of material and cracking. This is due to the expansive impact of the sulfate attacks. The composition of sulfate-based products, such as secondary ettringite, resulted in swelling. This caused micro-cracks and the devastation of the material. The realization of this stage in the small pores is accepted as the driving power for the expansion produced in the Portland cement samples, as in Figure 28 and Figure 29. The figures show the morphological changes in the pastes exposed to MS solution at 180 days. The Z1 mix microstructure of the pastes presented a compact and thick structure, with fewer cracks. It was composed of CSH, CH, and ettringite. The FA pozzolanic effect and BS fineness blocked the pores. Therefore, the MS did not penetrate deeply into the microstructure.

The strength at 365 days decreased by the range of 9–14% and 3–10% for groups Z and V, respectively, compared to the same reference mixture under normal conditions. This may be attributed to the dilution effect, which reduced the CH. In addition, exhausted by the pozzolanic reaction, the sulfate and magnesium ions reacted with CSH, leading to a magnesium silicate hydrate (MSH) with no cohesion. Ettringite is formed when sulfate reacts with CAH. It is an unstable compound that grows in size. As a result, the microstructure of the concrete began to crack.

The composition of gypsum results in a soft concrete surface that causes the loss of mass and strength, but secondary ettringite takes a greater volume than the foremost reactants, leading to expansion and concrete cracking. The resulting cracking allowed the transportation of sulfate ions into the concrete that speeded up the degradation of HWHPCs.

The results are attributed to the influence of FA pozzolanic, which is considered the most effective way of reducing the worsening influence of sulfate attack, as well as FS formation. The pozzolanic materials strengthen the concrete microstructure due to their particle size and can alter the chemical composition and hydration reactions, in particular the FA. With FA the, sulfate resistance, particularly MS, can be significantly increased.

### 4.6. Gamma Radiation Exposure

The linear attenuation coefficient (μ), half-value layer (HVL), tenth-value layer (TVL), and mean free path (mfp) of concrete mixes made with BS fine aggregate was estimated at a photon energy of 0.662 MeV for ^137^Cs. Table 3 displays the values of HVL and TVL of HWHPC mixes as an action of the BS ratio for various gamma energies released by ^137^Cs sources. The results indicated that Z1 with 15% BS is remarkably effective for shielding gamma rays. The attenuation values of BS concrete are more than those included in NS. It is clearly seen that the linear attenuation coefficient depends on the BS content used.

The HVL or TVL of a substance is utilized to denote the efficacy of gamma-ray shielding. HVL is the thickness at which an absorber reduces radiation to half its original intensity, while TVL is the thickness where an absorber decreases the radiation to one-tenth of its original intensity [57]. The HVL and TVL values of a HWHPC mix decline as the BS ratio for ^137^Cs increases. The lower the value of the HVL and TVL, the better the radiation shielding materials are at increasing the BS ratio. Nevertheless, the HVL and TVL values for HWHPC mixes incorporating BS fine aggregate were less than the Z0 incorporating LS. The results also revealed that the values of HVL and TVL were directly related to the BS concrete density, so Z1 had lower HVL and TVL values than Z6.

### 4.7. Correlation Analysis between Different Properties of Heavyweight High Performance Concrete

#### 4.7.1. Relationship between Mass Loss and Compressive Strength 

Under the same various exposure conditions, the association between mass loss and compressive strength is shown in this section for group Z mixtures. The correlation coefficient (*r*) was calculated using Microsoft Excel’s “Pearson” function [58], as shown in Equation (7).

(7) r=n∑XiYi−∑Xi∑Yi n∑Xi^2−∑Xi^2n∑Yi^2−∑Yi^2

The estimated *r* values fall within the range of (−1, 1). An *r* = 1 indicates that the correlation is fully linear; *r* = −1 indicates that the correlation is inversely linear; and *r* = 0 indicates that the pairs of values are fully independent. The degree of correlated closeness is then classified as medium if 0.3 < *r* < 0.5; significant if 0.5 < *r* < 0.7; high if 0.7 < *r* < 0.9; and extremely high if 0.9 < *r*. A high correlation indicates a significant association between two or more variables, whereas a low correlation indicates that the variables are hardly related. 

**Figure 30:**
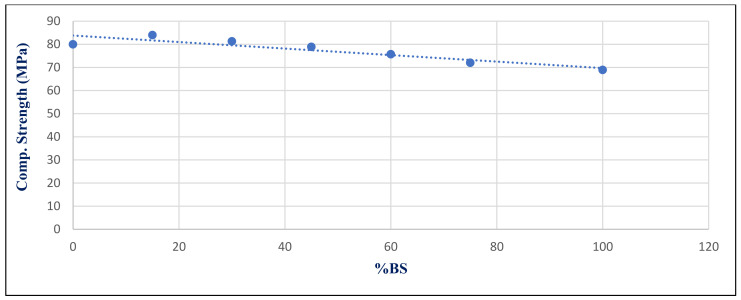
Correlation relation between compressive strength at a 750 °C fire exposure and %BS for group Z at day 180.

**Figure 31:**
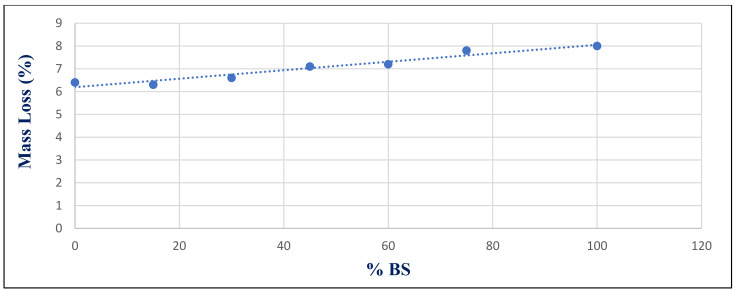
Correlation relation between %BS and %Mass loss at 750 ^o^C for group Z at day 180.

Figure 30 depicts the relationship between compressive strength and percent BS replacement for group Z after 180 days of 750 °C fire exposure. Under 750 °C, there was a strong link between the BS ratio and compressive strength, with a correlation coefficient of −0.917. Figure 31 shows the relationship between mass loss and BS replacement ratio for group Z at 180 days after a 750 °C fire. With a correlated coefficient of 0.969, it shows a substantial association between %BS and %mass loss. Owing to the microcline formed, the strength deteriorated and mass loss increased, as illustrated in Figure 20.

**Figure 32:**
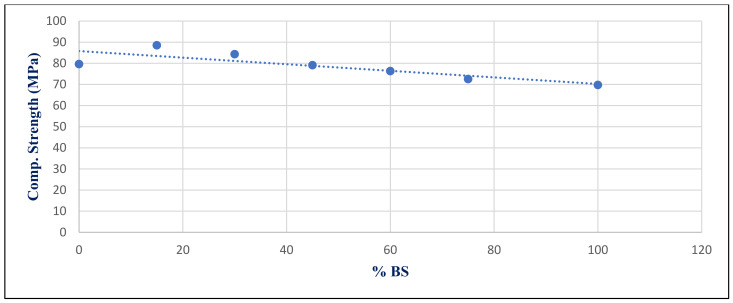
Correlation relation between compressive strength under sulfate exposure conditions and %BS for group Z at day 365.

**Figure 33:**
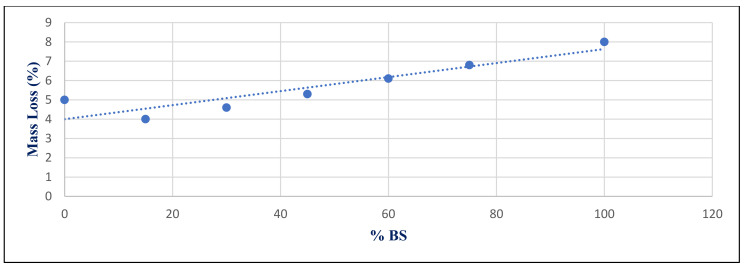
Correlation relation between %BS and %Mass loss under sulfate exposure conditions for group Z at day 365.

Figure 32 shows the inverse relation between compressive strength under sulfate exposure condition with %BS replacement for group Z at 365 day, with a correlation coefficient of −0.834. Figure 33 shows the relation between mass losses under sulfate exposure conditions with %BS replacement for group Z at 365 day. It illustrates the significant correlation between %BS and %mass loss, with a correlation coefficient of 0.917.

The statistical group included seven values that represented the input data used to compute the correlation coefficients, as illustrated in Figure 30, Figure 31, Figure 32 and Figure 33. The calculation took into account all of the measured data. Table 4 and Table 5 show the relationship between compressive strength and mass loss ratio under various exposure conditions. Under varied exposure conditions, this revealed an inversely significant relationship between mass loss and compressive strength.

#### 4.7.2. Relationship between Density and Compressive Strength under Fire Exposure Condition at Day 28 

**Figure 34:**
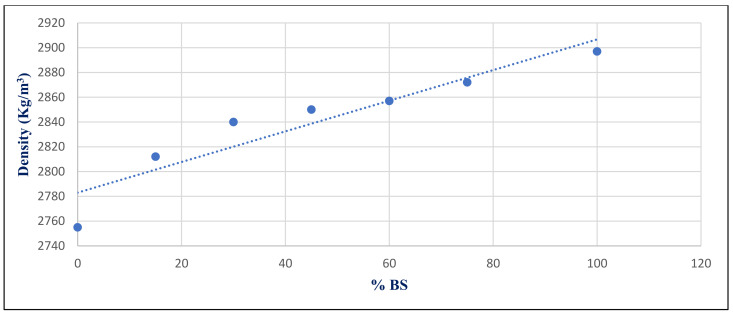
Correlation relation between %BS and density for group Z at day 28.

**Figure 35:**
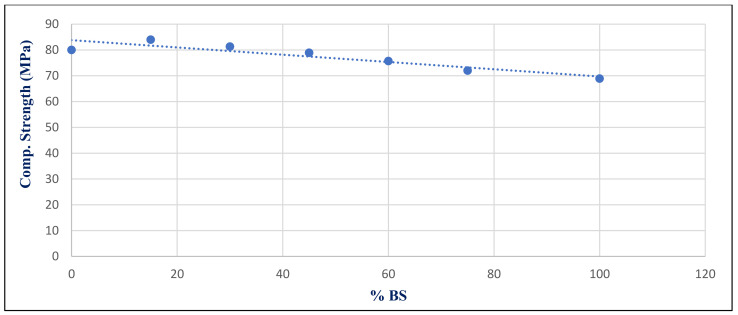
Correlation relation between %BS and compressive strength for group Z at 750 °Cat day 28.

Figure 34 shows the relationship between density and BS replacement ratio for group Z at 28 days after a 750 °C fire. With a correlation coefficient of 0.9378, it showed a very good association between %BS and density. Figure 35 illustrates the inverse relation between compressive strength under 750 °C exposure conditions with %BS replacement for group Z at day 28, with a correlation coefficient of −0.7446. 

**Figure 36:**
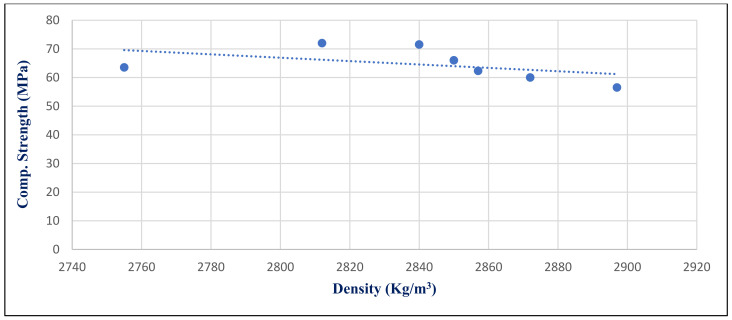
Correlation relation between density and compressive strength for group Z at 750 °Cat day 28.

Figure 36 illustrates the inverse medium relation between HWHPC density and compressive strength under 750 °C exposure conditions at day 28, with a correlation coefficient −0.4724. Generally, the HWHPC density has a medium inverse influence on compressive strength under various exposures, because increasing the mineral content in the microstructure forms deteriorated compounds, such as microcline.

## 5. Conclusions

In this paper, the effect of new fine aggregate shielding construction materials, namely black sand (BS), on HWHPC mechanical, shielding, and durability properties were studied. The following conclusions were drawn from the obtained results:

- BS is a promising concrete shielding material that is rich in a specific mineral that improves HWHPC properties concertedly, particularly elevated temperature resistance.
- Using BS increased the average density of HWHPC by 23% more than the control mixture, X0.
- Using 15% BS with 10% FA (Z1) is the optimum ratio that improves all HWHPC properties.
- The mass loss of Z1 was recorded as −4%, −5.1%, and −6.3%, compared to −7.3%, −11.2%, and −18.5% for X0 at 250 °C, 500 °C, and 750 °C exposure conditions.
- The mass loss of Z1 recorded +1.5%, −2.5%, and −4%, compared to +1.1%,−9%, and −12% for X0 at 90, 180, and 365 days with sulfate exposure conditions.
- The compressive strength under normal, 250 °C, 500 °C, and 750 °C exposure conditions of Z1 was 132%, 134%, 135%, and 181% higher than X0, and 106%, 108%, 106%, and 105% higher than Z0 at 180 days.
- The compressive strength under sulfate exposure conditions of Z1 was 139%, 178%, and 206% higher than X0 at 90, 180, and 365 days.
- Z1 showed significant shieling attenuation results, despite not using HWA as a coarse aggregate.

## 6. Recommendation

Future studies should focus on the following subjects:

- Study group Z durability properties, especially fire resistance at temperatures over 750 °C.
- Study the effects of using HWA such as serpentine, magnetite, and iron slag with greyish-yellow BS on HWHPC shielding attenuation properties, especially at elevated temperatures.

## Figures and Tables

**Table 1:** Oxide composition of binder materials and black sand, wt% [41].

	Cement	Black Sand	Fly Ash
SiO_2_	21.25	60.90	60.27
Al_2_O_3_	4.67	4.41	27.99
Fe_2_O_3_	3.65	9.71	4.54
CaO	61.8	7.95	1.16
MgO	2.72	2.75	0.37
SO_3_	3.06	0.27	0.35
Na_2_O	0.18	0.67	0.15
K_2_O	0.12	0.27	0.98
TiO_2_	-	6.41	-
P_2_O_5_	-	0.36	-
Cl-	-	0.39	-
Cr_2_O_3_	-	0.27	-
MnO	-	0.43	-
ZrO_2_	-	0.53	-
L.O.I	2.50	4.52	0.91
Total	99.95	99.83	96.72

**Table 2:** Mixes proportion (kg/m^3^).

Mix Groups	Mix	PC	FA	W	NS	BS	D	SP
Control	X0	500	-	150	892.9	-	892.9	7.5
	Z0	450	50	140	898.4	-	898.4	
	V0	425	75	135	901.1	-	901.1	
Z	Z1	450	50	140	752.4	132.8	885.1	
	Z2			155	615.0	263.5	878.5	
	Z3			160	479.5	392.4	871.9	
	Z4			165	346.0	519.0	865.2	
	Z5			170	214.6	644.0	858.6	
	Z6			175	-	852.0	852.0	
V	V1	425	75	145	754.7	133.2	887.9	
	V2			150	616.9	264.4	881.2	
	V3			155	481.0	393.6	874.9	
	V4			160	347.0	520.8	868.0	
	V5			171	213.5	640.5	854.0	
	V6			176	-	846.3	846.3	

**Table 3:** The HWHPC radiation shielding properties.

Mix	γ—Sources	%BS	μ—(cm^−1^)	HVL (cm)	TVL (cm)	mfp (cm)
Z0	^137^Cs	0%	0.1965	3.527	11.718	5.089
Z1		15%	0.2046	3.388	11.254	4.887
Z2		30%	0.1985	3.492	11.60	5.037
Z6		100%	0.1980	3.50	11.628	5.050

**Table 4:** The correlation coefficient between %BS, compressive strength, % mass loss at 750 °C fire exposure at 180 days for group Z.

Correlation Parameters	%BS	% Mass Loss at 750 °C	Comp. Fire at 750 °C
%BS	1	0.969	−0.917
% Mass loss under 750 °C	0.969	1	−0964
Comp. fire under 750 °C	−0.917	−0.964	1

**Table 5:** The correlation coefficient between %BS, compressive strength, % mass loss with sulfate exposure at 365 days for group Z.

Correlation Parameters	%BS	% Mass Loss under Sulfate Exposure	Comp. Fire under Sulfate Exposure
%BS	1	0.917	−0.834
% Mass loss under 750 °C	0.917	1	−0968
Comp. fire under 750 °C	−0.834	−0.968	1

## Data Availability

Data is contained within the article.
